# Language, literacy, and sensory impairments and missing cognitive test scores in the Harmonized Cognitive Assessment Protocol of the China Health and Retirement Longitudinal Study

**DOI:** 10.1007/s40520-025-03039-y

**Published:** 2025-05-12

**Authors:** Alden L. Gross, Ying Liu, Yuan S. Zhang, Yaohui Zhao, Chihua Li, Erik Meijer, Jinkook Lee, Lindsay C. Kobayashi

**Affiliations:** 1 Department of Epidemiology, Johns Hopkins Bloomberg School of Public Health, 616 N. Wolfe St., Baltimore, MD 21205 USA; 2 Center On Aging and Health, Johns Hopkins University, 2024 E. Monument St., Baltimore, MD 21205 USA; 3 Center for Economic and Social Research, University of Southern California, 635 Downey Way, Los Angeles, CA USA; 4 Department of Sociomedical Sciences, Mailman School of Public Health, Columbia University, New York, NY USA; 5 Robert N. Butler Columbia Aging Center, Mailman School of Public Health, Columbia University, New York, NY USA; 6 Department of Economics, Peking University, Beijing, China; 7 Center for Social Epidemiology and Population Health, Department of Epidemiology, University of Michigan School of Public Health, Ann Arbor, MI USA; 8 Survey Research Center, University of Michigan Institute for Social Research, Ann Arbor, MI USA; 9 Department of Economics, University of Southern California, 635 Downey Way, VPD, Los Angeles, CA USA

**Keywords:** Missing data, Differential item functioning, Population survey, Cognitive performance, Gerontology, China

## Abstract

**Background:**

The potentially biasing impacts of low language fluency, illiteracy, and sensory impairments on cognitive test performance are unknown, which may have implications for understanding their roles in cognitive decline and dementia.

**Aims:**

We investigated effects of these features on cognitive test item completion and performance among older adults in China, a multilingual country with high prevalence of illiteracy and sensory impairment.

**Methods:**

We used cognitive test data from the Harmonized Cognitive Assessment Protocol of the China Health and Retirement Longitudinal Study conducted in 2018 (N = 9755, age 60 + years). We first tested associations of fluency in spoken Mandarin, literacy, and sensory impairment (hearing and vision) with missingness of cognitive items. We then tested for differential item functioning (DIF) in observed cognitive items by these features.

**Results:**

We observed high levels of missing data in most cognitive test items – on average 13% and as high as 65%. Low fluency in spoken Mandarin, illiteracy, and impairments in hearing and vision were each associated with greater odds of missingness on nearly all tests. Partly because of differential missingness, there was minimal evidence of DIF by these features in items in which we expected a priori to find DIF (e.g., repetition of a spoken phrase among those with hearing impairment). Several cognitive test items exhibited statistically significant DIF, however there was minimal evidence of meaningful DIF.

**Conclusions:**

Differential missingness in cognitive items by spoken language, literacy, and sensory impairments is potentially more of an inferential threat than measurement differences in test items.

**Supplementary Information:**

The online version contains supplementary material available at 10.1007/s40520-025-03039-y.

## Introduction

China is a linguistically diverse country with at least nine major oral languages, including standard Mandarin and its seven major dialects, as well as Cantonese and Hunanese [1]. However, many older adults in China do not speak standard Mandarin [2]. Further, illiteracy is estimated at 37% among older adults in China due to a lack of access to formal education historically [3, 4]. The Harmonized Cognitive Assessment Protocol (HCAP) of the China Health and Retirement Longitudinal Study (CHARLS) represents older adults from the world’s most populous country in an upper-middle income economic setting [5, 6]. HCAP is an international initiative, supported by the US National Institute on Aging, to facilitate collection of harmonized cognitive data across international partner studies of the HRS [7]. Interview materials for the CHARLS-HCAP were prepared and administered assuming fluency in spoken and written Mandarin (Putonghua), the most common dialect of Mandarin Chinese. In addition to a diversity of languages and illiteracy in China, among Chinese adults aged > 70 years there is also a high prevalence of uncorrected visual impairment estimated 16% among men and 22% among women [8] and hearing impairment (estimated 59%) [9].

For the CHARLS-HCAP to be a robust internationally harmonized population data source on the cognitive function of the older population in China, it is imperative to ensure its measures are administered and scored in a fashion that is unbiased and appropriate to the language, literacy, and sensory capacities of its population. It is possible that CHARLS-HCAP participants who did not speak Mandarin, who were illiterate, and/or who had uncorrected visual impairment at the time of HCAP administration may have systematically lower scores on cognitive test items than those who spoke Mandarin, were literate, and did not have any visual impairment. If this is the case, lower scores may be due to a lack of ability to complete the HCAP test battery as administered, rather than due to lower cognitive ability per se.

Previous research in other study populations suggests illiteracy can be a biasing factor in the measurement of cognitive function. Literacy is thought to be a mechanism through which education protects against dementia, and the promotion of reading and writing skills across the life course may have positive effects on the brain [10–13]. However, older adults who are illiterate may perform worse on certain tests of cognitive function that are sensitive to literacy levels, such as writing a complete sentence, due to a lack of literacy skills, rather than their underlying level of cognitive function [14, 15]. Previous research amongst older adults with varying literacy levels has found differential item functioning (DIF) of cognitive test items by literacy status, such as the cognitive screening assessment in the Longitudinal Aging Study in India [16]. In previous research using the Baltimore Longitudinal Study of Aging (BLSA) and Atherosclerosis Risk in Communities Neurocognitive Study (ARIC-NCS), we observed DIF according to visual impairment in performance on cognitive tests [17]. These findings were in non-representative highly educated, English-speaking samples, such that participants with sensory impairment may have had a better ability to compensate for impairment than those in the general population. Thus, the potentially biasing effects on HCAP performance by vision and hearing impairment status in China are unknown.

Interview materials for the CHARLS-HCAP were not adapted to account for non-Mandarin language fluency, illiteracy, or sensory impairment. The potentially biasing impacts of this lack of standardized translations or adaptations of the CHARLS-HCAP test items are unknown. The purpose of the present study was to determine if and how cognitive test items in the CHARLS-HCAP may be biased by fluency with spoken Mandarin, literacy, and sensory (visual and hearing) impairment. In this context, we use the term *bias* to describe performance on cognitive tests that is conditional on extraneous features outside of one’s true level of cognitive function, holding cognition constant.

## Methods

### Sample

CHARLS is an ongoing longitudinal study of a nationally representative sample of > 10,000 Chinese residents aged 45 and older and > 7,000 of their spouses [6]. Interviews started in 2011 (Wave 1), with follow-ups in 2013 (Wave 2), 2015 (Wave 3), 2018 (Wave 4), and 2020 (Wave 5). Participants provide data on demographic, socioeconomic, health, and behavioral factors through in-person interviews by trained fieldworkers, in addition to undergoing physical and cognitive assessments and providing biomarker data. Prior to 2018, CHARLS used a brief cognitive assessment harmonized with that used in the US Health and Retirement Study (HRS). In 2018 (Wave 4), the CHARLS administered the HCAP to all participants aged ≥ 60 years [5]. The survey was completed in accordance with Helsinki Declaration.

### Variables

#### Cognitive testing

The CHARLS-HCAP battery was translated into Mandarin from the English-language version of the HCAP battery which was administered in the HRS in 2016. Details of the battery are available elsewhere [7, 18]. The CHARLS-HCAP battery included 32 cognitive test items including orientation to time and place, word list recall, story recall, semantic fluency, and object naming (Table 1). To maintain a high degree of harmonization with the HRS-HCAP, this translation was strict in nature without content adaptations for the cultural context of China or for individuals with illiteracy or sensory impairment.

**Table 1:** Sample characteristics of CHARLS-HCAP (N = 9755)

Characteristic	Mean (SD) or N (%)	Range	N (%) Missing
Age, years, mean (SD)	68.5 (6.5)	60, 108	
Female sex, N (%)	4960 (50.8)		
Formal schooling, N (%)			
None or Early Childhood Education	4909 (50.3)		
Primary education	2355 (24.1)		
Lower secondary	1562 (16.0)		
Upper secondary	929 (9.5)		
Place of residence, N (%)			
Rural	7203 (73.8)		
Urban	2552 (26.2)		
Language group, N (%)			
Confirmed Mandarin	3917 (40.2)		
Likely Mandarin	563 (5.8)		
Likely non-Mandarin	5275 (54.1)		
Illiterate, N (%)	3491 (35.9)		
Possible hearing impairment, N (%)	2862 (29.3)		
Possible vision impairment, N (%)	3002 (30.8)		
Cognitive test items, mean (SD)			
Orientation—day of month	0.64 (0.48)	0, 1	775 (7.9)
Orientation—month	0.88 (0.32)	0, 1	231 (2.4)
Orientation—year	0.72 (0.45)	0, 1	777 (8.0)
Orientation—day of week	0.62 (0.49)	0, 1	925 (9.5)
Orientation—state	0.86 (0.35)	0, 1	519 (5.3)
Orientation—county	0.88 (0.32)	0, 1	375 (3.8)
Orientation—city	0.94 (0.25)	0, 1	205 (2.1)
Orientation—season	0.72 (0.45)	0, 1	622 (6.4)
Orientation—floor	0.90 (0.30)	0, 1	585 (6.0)
Orientation—address	0.94 (0.23)	0, 1	304 (3.1)
CERAD immediate recall	12.04 (6.26)	0, 30	1765 (18.1)
3-word delayed recall	2.08 (1.00)	0, 3	1241 (12.7)
CERAD delayed recall	3.54 (2.67)	0, 10	2079 (21.3)
CERAD recognition	17.10 (3.50)	0, 20	2141 (21.9)
3-word immediate registration	2.33 (0.93)	0, 3	690 (7.1)
Number series	7.49 (2.85)	0, 15	3685 (37.8)
Serial 7 s	3.31 (1.61)	0, 5	3165 (32.4)
Animal fluency	11.73 (4.83)	0, 50	1632 (16.7)
What do you use to cut paper	0.95 (0.22)	0, 1	913 (9.4)
Naming a watch	0.89 (0.31)	0, 1	147 (1.5)
Naming a pencil	0.91 (0.29)	0, 1	146 (1.5)
Name a cactus	0.44 (0.50)	0, 1	6334 (64.9)
Name elbow	0.87 (0.33)	0, 1	201 (2.1)
Write a sentence	0.64 (0.48)	0, 1	4919 (50.4)
Phrase repetition	0.43 (0.49)	0, 1	1631 (16.7)
Read and follow command (Close your eyes)	0.82 (0.38)	0, 1	816 (8.4)
What do you do with a hammer	0.91 (0.28)	0, 1	326 (3.3)
Two-step instructions	0.79 (0.40)	0, 1	862 (8.8)
Where is the local market?	0.74 (0.44)	0, 1	732 (7.5)
Three-step instructions	2.10 (0.95)	0, 3	454 (4.7)
Interlocking pentagons	0.53 (0.50)	0, 1	1980 (20.3)
Name the president	0.94 (0.24)	0, 1	1143 (11.7)

#### Spoken language/dialect

Reliable data on the languages spoken by participants were not collected in CHARLS. The language or dialect used by interviewers during each interview was recorded as Mandarin (Standard Chinese or Putonghua) (47% of the CHARLS-HCAP sample), a local dialect (51%), or another dialect (2%). We used this information, coupled with the dominant language in the region derived based on participants’ county of residence and the publicly available Language Atlas of China [19], as well as information about whether a local translator was used during the interview, to create categories of language likely spoken by participants. We categorized participants as confirmed Mandarin speakers (if the interview was conducted in Mandarin, no translator was used, and the dominant language in the region is Mandarin), likely Mandarin speakers (if the interview was conducted in Mandarin, however a translator was used and/or the dominant language in the region is not Mandarin), and likely non-Mandarin speakers (if the interview was conducted using a local dialect or other dialect, and/or a translator was used, and/or the dominant language in the region is not Mandarin).

#### Illiteracy

Participants self-reported their literacy (yes or no). When this information was missing (for 47% of participants), we assumed respondents with no formal schooling and no sishu (private schooling) were illiterate.

#### Hearing impairment

We classified participants as possibly hearing impaired if they self-reported being deaf or half deaf (您是否有聋或半聋) in any CHARLS wave from 2011–2018, self-rated their hearing (with correction if participants usually wore hearing aids) as poor (不好) on a 5-point Likert scale in the 2018 wave, or if the interviewer reported participants’ poor hearing during the 2018 cognitive interview. Participants were also classified as possibly hearing impaired if they refused the word list learning or semantic fluency tasks of the 2018 HCAP battery due to being deaf or poor of hearing.

#### Vision impairment

We classified participants as possibly visually impaired or not, based on responses to available self-report questions. Participants who reported being blind or half blind in any CHARLS wave from 2011 to 2018 (您是否有失明或半失明), reported being legally blind when asked about prescription glasses or corrective lens wearing in 2018, or self-rated their near vision as poor (不好) on a 5-point Likert scale in 2018 were classified as possibly vision impaired. Participants were also coded as possibly vision impaired if interviewers observed evidence of poor eyesight during the 2018 HCAP interview.

#### Control variables

Key covariates used to characterize the sample and as adjustment variables in DIF and missingness analyses included age in years, sex (male, female), educational attainment, urbanicity (urban/rural residence), and ethnic minority status. Educational attainment was operationalized into categories of none or early childhood education, primary or private (Sishu) education, lower secondary, or upper secondary or college based on the International Standard Classification of Education [20]. Ethnic minority status was defined as non-Han. We included urbanicity and ethnic minority status as additional covariates to account for potential confounders predictive of survey participation (but not necessarily cognition). Particularly, lower social engagement and trust in institutions are linked to lower willingness to participate in surveys [21], and such factors may vary by urbanicity and ethnic minority status in China [22].

### Statistical analyses

We computed descriptive statistics for each variable. Next, we evaluated differential missingness in cognitive test items by fluency in spoken Mandarin, illiteracy, and sensory impairments. Third, we tested for DIF in cognitive test items by these features.

#### Characterization of missingness in cognitive test data

We classified cognitive test items as missing for a respondent if the item was system missing or missing for any stated reason (Don’t know, Refused, Not assessed). Conventional practice is to classify responses of “don’t know” as incorrect with respect to cognitive testing because it can be assumed the participant understood the question (otherwise they could refuse) but does not know how to answer the question. We chose not to code “Don’t know” as incorrect because missing data codes in the CHARLS-HCAP were inconsistent across items and across cognitive testlets, possibly because training and instructions to interviewers were unclear in some cases. We also did not differentiate missingness due to an item being not assessed from other types of missingness because that missingness code was applied inconsistently across variables in the data. Moreover, respondents may not have communicated their reasons clearly during interviews. For example, missingness among the 10 questions assessing orientation to date and time was coded as “not assessed” in 2–9% of cases or as system missing in other cases, however only in 33 cases (0.33%) were all the orientation items consistently coded together as “not assessed.” As another example, of N = 1765 participants (18%) missing data on the CERAD sum of three immediate recall trials, N = 452 had 0 recall for trial 1 but N = 1313 were coded as system missing on all trials – with no indication of whether participants refused the test, didn’t know any words, or were not administered the test –and the majority of these N = 1313 participants completed the rest of the HCAP battery.

To evaluate predictors of missingness in cognitive test items, we estimated multivariable logistic regressions of missingness indicators for each cognitive test item on indicators for spoken language fluency (likely Mandarin, likely non-Mandarin, confirmed Mandarin), illiteracy, and impairment in hearing and vision. Logistic regression models were adjusted for sex, urbanicity, and ethnic minority status.

#### Differential item functioning (DIF)

**Fig. 1:**
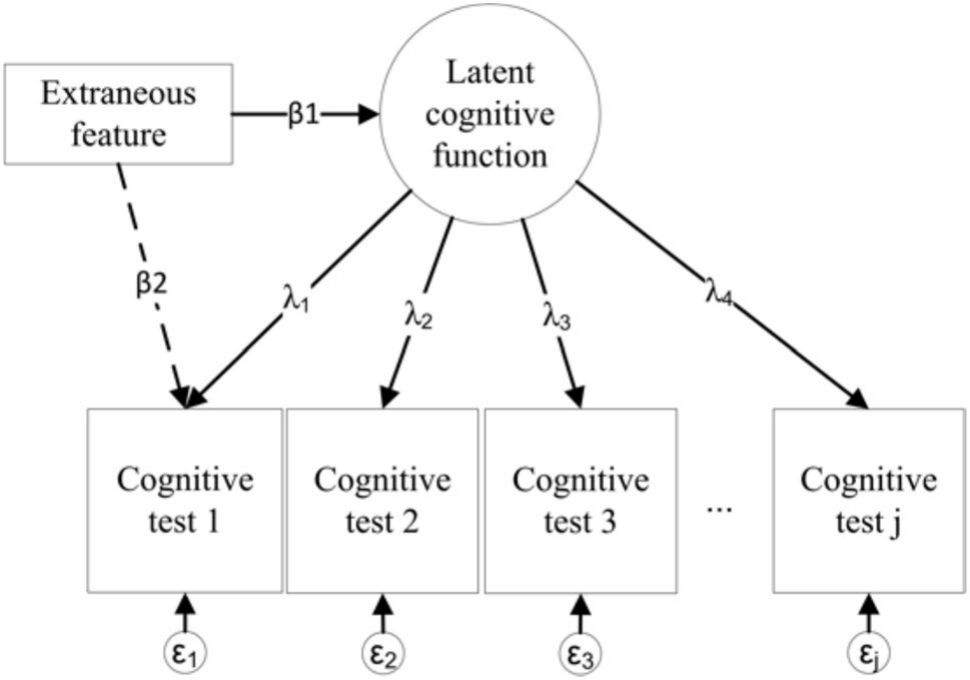
Path diagram to illustrate detection of Differential Item Functioning using MIMIC modeling. This figure shows a path diagram for a Multiple Indicators, Multiple Causes (MIMIC) model. Squares and rectangles indicate observed variables. Circles represent unobserved latent variables that can be inferred from items (cognitive test items 1 through J). This figure describes a measurement model for latent cognitive function informed by cognitive test items 1 through J (see Table 1 for a list of cognitive test items used in this study), wherein the factor loadings (λ_j_) indicate the strength of the relation between each indicator and the latent trait. Each item has a residual error (measurement error), ε_j_. Each cognitive test item, alongside latent cognitive function, is sequentially regressed on an extraneous feature (e.g., spoken language, literacy, hearing or vision impairment; paths β_2_ and β_1_, respectively). The magnitude of path β_2_ indicates the association of the extraneous feature with the cognitive test item, controlling for underlying latent cognitive function

We assessed DIF in item threshold parameters in the CHARLS-HCAP via the multiple indicators multiple causes (MIMIC) framework [23, 24]. This framework enables us to disentangle underlying cognitive function from extraneous features such as language or dialect of interview, literacy, or sensory impairment. If there are no differences in a cognitive test item attributable to these features, item scores should vary as a function *only* of underlying cognitive function (path λ_j_ in Fig. 1), and not as a function of the extraneous feature (path β_2_), after controlling for the relationship between the extraneous feature and cognitive function (β_1_) and other covariates. Item threshold parameters indicate the level of difficulty of an item, relative to other items along the latent cognitive function factor; DIF in thresholds is consistent with the notion of systematic error, or test bias [25]. The MIMIC model we used for this study is a confirmatory factor analysis in which indicators (e.g., a cognitive test item) and the latent factor (e.g., cognitive function factor) are simultaneously regressed on a predictor (e.g., language fluency, literacy, or vision impairment) (paths β_2_ and β_1_ in Fig. 1, respectively) [26].

Path β_2_ in Fig. 1, which indicates differences in underlying cognitive ability by an extraneous predictor or feature, is expressed as an odds ratio, and can be interpreted as the magnitude of DIF in an item. We considered odds ratios outside the range of 0.66 to 1.5 to indicate DIF of a non-negligible magnitude [18, 27].

#### Adjustment for background characteristics in MIMIC models

MIMIC models can be adjusted for background characteristics in addition to the grouping variable of interest. Most MIMIC models in the present study are further adjusted for age, sex, and educational attainment; an exception was for illiteracy, in which we did not adjust for education given its collinearity with literacy.

#### Salient DIF

DIF, whether negligible or non-negligible, can be corrected by retaining effects of the feature on the item, thus removing differences in cognitive function attributable to that feature. However, prior to correcting cognitive factors for statistically significant DIF in an item, we ascertained DIF impact on factor scores by calculating and comparing the difference between a non-DIF adjusted factor score and a factor score that was adjusted for DIF by retaining effects of the feature on the indicator. Common practice is to flag participants with non-DIF-adjusted and DIF-adjusted scores differing by > 0.30 SD units across the two sets of factor scores as impactful DIF [18].

#### Selection of anchor items for DIF testing

To prevent incorrect inferences, it is crucial to select anchor items prior to estimating a MIMIC model, which are test items considered to be free of DIF. We used a sequential-free baseline approach to empirically select anchor items [28, 29]. This approach involves three steps for each feature of interest. First, we looped through each cognitive test item in a series of confirmatory factor analyses to test for differences in thresholds by the feature. This step alone will lead to inflated type I error rates (i.e., more DIF-free items would be wrongly classified as DIF), however, this is not concerning for the present analysis because it provides a more conservative set of candidate anchors. Second, from the set of candidate anchors not identified as having DIF in the first step, we selected the cognitive test items with the highest factor loadings (i.e., the most discriminating items) as anchor items. Third, we tested for DIF among the set of anchor items for each feature; by dropping items from the list of anchors that had even small amounts of DIF, we could be certain we had identified strong anchor items.

#### Missing data handling in MIMIC models

MIMIC models are based on confirmatory factor analysis, which here uses full information maximum likelihood estimation that assumes missingness is conditionally independent of the unobserved variables in the analysis, given observed variables 30). This missing at random (MAR) assumption becomes questionable when the missingness is driven by the respondent and the missing mechanism is unknown or cannot be reasonably assumed. For instance, when missingness in cognitive test items is related to cognitive test performance and to the grouping feature itself, there can be systematic error in DIF detection [30].

## Results

### Descriptive characteristics

Among N = 9755 participants, N = 3917 (40.1%) were confirmed Mandarin speakers, N = 563 (5.8%) were likely Mandarin speakers, and N = 5275 (54%) were likely non-Mandarin speakers (Table 1). The unweighted prevalence of illiteracy, hearing impairment, and vision impairment was N = 3491 (35.9%), N = 2862 (29.3%), and N = 3002 (30.8%), respectively.

### Missingness in cognitive test data

As shown in Table 1, the percentage of missing data in cognitive test items in the CHARLS-HCAP ranged from 1.5% (naming a pencil, naming a watch) to 65% (cactus). The median amount of missingness was 8.2%. Items with the most missingness included cactus naming (65%), writing a sentence (50%), number series (38%), serial 7 s (32%), and items for CERAD word list recall (18–22%).

**Fig. 2:**
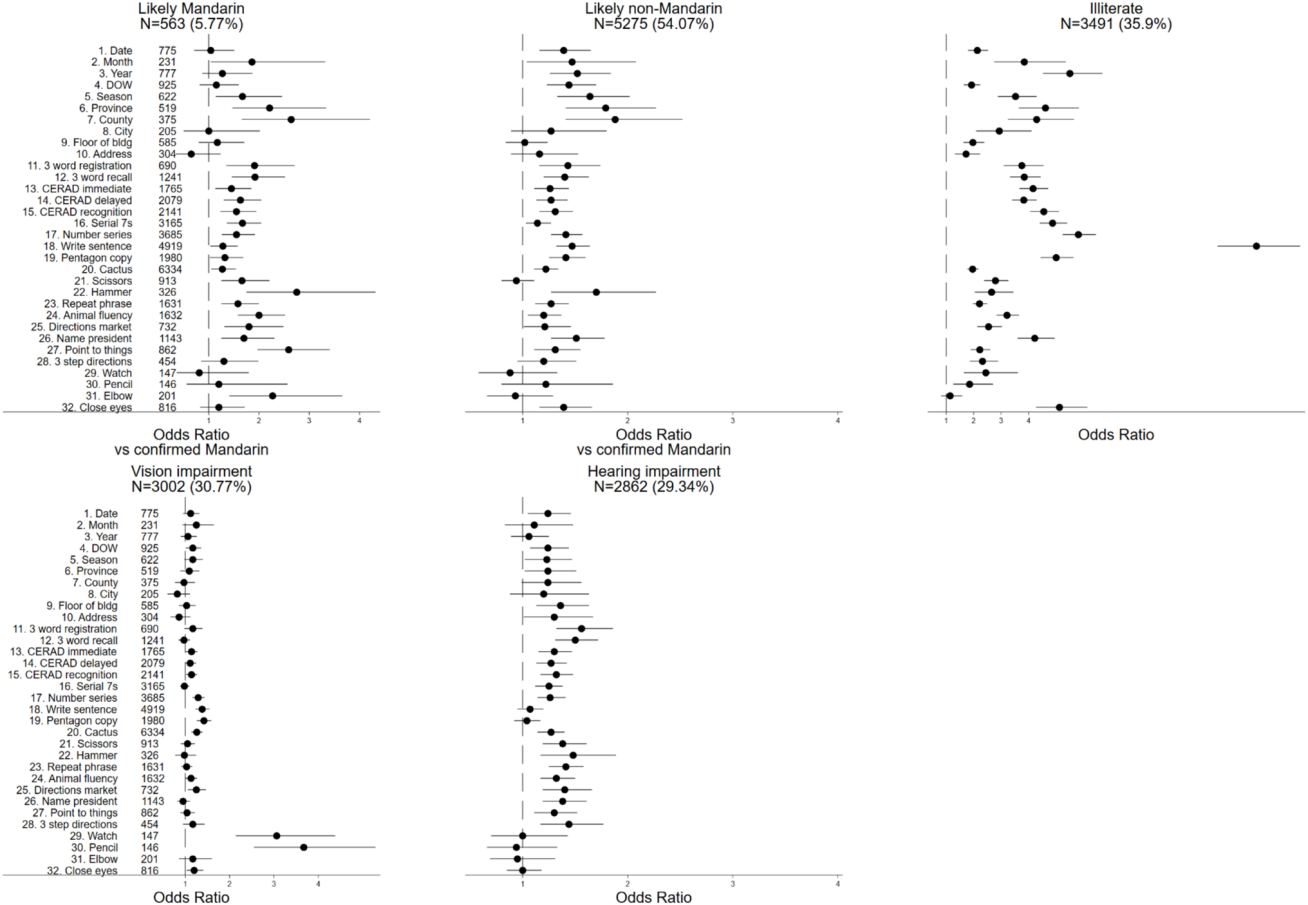
Odds of missingness by language, literacy, and sensory impairment: Results from CHARLS-HCAP (N = 9755). Odds ratios are from multivariable logistic regressions of missingness indicators for each cognitive test item on indicators for spoken language (likely Mandarin, likely non-Mandarin, confirmed Mandarin), literacy, and impairment in hearing and vision. Models were adjusted for sex, urbanicity, and ethnic minority status

Spoken language fluency, illiteracy, hearing impairment, and vision impairment were each associated with significantly elevated odds of missingness for 26, 31, 22, and 11 cognitive test items, respectively (Fig. 2). Certain patterns emerged from this analysis. For example, illiterate participants were 12 times as likely to be missing the “write a sentence” item as literate participants, and visually impaired participants had an elevated odds of missingness for naming a watch or a pencil (which are presented visually in pictures on a tablet). The odds of missingness for CERAD immediate, delayed, and recognition tasks was higher among likely Mandarin and likely non-Mandarin (compared to confirmed Mandarin) speakers, illiterate, hearing impaired, and visually impaired participants.

### DIF by language fluency

We evaluated DIF in cognitive test items between likely Mandarin speakers vs confirmed Mandarin speakers, and between likely non-Mandarin speakers vs confirmed Mandarin speakers. For the former contrast, we identified 21 anchor items; of the remaining 11 items, 8 showed DIF that was statistically significant but negligible in magnitude (Supplemental Table 1). Of the 8 items, 3 items involved naming objects while another three measured orientation to time. The item with the largest magnitude of DIF was elbow naming (OR 0.66; 95% CI 0.58, 0.75); ORs below 1.0 indicate that, controlling for underlying cognitive performance and background characteristics, the item is more likely to be correct among confirmed Mandarin speakers than likely Mandarin speakers. For the latter contrast, we identified 17 anchor items; of the remaining 15 items, 1 showed DIF that was statistically significant but negligible in magnitude (orientation to city). Despite statistically significant DIF, there was no evidence of salient or meaningful DIF by spoken language fluency (Supplemental Table 2). Notably, models failed to identify significant and non-negligible DIF in any word recall item or naming item (e.g., watch, pencil, cactus, elbow) by spoken language ability, items which one might expect to show DIF.

### DIF by illiteracy

Of the 32 cognitive test items, MIMIC models identified DIF by illiteracy in 16 items (Supplemental Table 1). Items with the largest magnitude of DIF were orientation to year (OR: 0.59; 95% CI: 0.55, 0.64) and writing a sentence (OR: 0.58; 95% CI: 0.50, 0.67). Despite considerable statistically significant DIF in cognitive test items, there was minimal evidence of salient DIF (Supplemental Table 2). Of note, models failed to identify DIF by literacy in an item requiring participants to read and follow a command (close your eyes) or in any word recall item.

### DIF by hearing impairment

Of the 32 cognitive test items, MIMIC models identified significant DIF in 9 items by possible hearing impairment, all of which were negligible (Supplemental Table 1). There was no evidence for salient DIF in cognition by hearing impairment status (Supplemental Table 2).

### DIF by vision impairment

Of the 32 cognitive test items, MIMIC models identified significant DIF in 7 items by possible vision impairment, all of which were negligible (Supplemental Table 1). There was no evidence for salient DIF in cognition by vision impairment status (Supplemental Table 2).

## Discussion

This study’s goal was to determine if and how cognitive test items in the CHARLS-HCAP may be biased by fluency with spoken Mandarin, illiteracy, and sensory impairment. Although we found minimal evidence of measurement differences (e.g., DIF), we noted large amounts of differential missing data that may have precluded the ability of statistical methods to detect such measurement differences. Differential missingness in cognitive test items by language of test administration, illiteracy, and sensory impairments is thus potentially more of a threat to inferences and generalizability of findings than measurement differences in cognitive test items detectable via DIF testing. This knowledge can inform the design and implementation of future epidemiologic studies, and it also has clinical implications for healthcare workers, policymakers and stakeholders. Results from this study underscore the necessity of translating and adapting cognitive test items based on one’s target population for linguistic, literacy, and sensory impairment needs. Additionally, it is important to reduce missing data during data collection, carefully document mechanisms for missing data, as well as objectively assess language/dialect of test administration, illiteracy, and sensory function in older participants who undergo cognitive assessments in population-based studies.

Previous research has examined DIF across language translations of other cognitive batteries, often finding evidence for DIF in these translated batteries [31–34]. There is also some evidence that bilingual older adults perform worse on cognitive tests that are administered in their secondary language [35, 36]. We inferred most participants in CHARLS-HCAP were not tested in their primary language, supported by the facts that we could only be confident that about 40% of participants spoke Mandarin, and interview materials were translated into other languages and dialects by interviewers in an ad hoc fashion. As important as linguistic considerations are cultural considerations of test items. For example, the cactus item (i.e., what is a prickly green plant that grows in the desert?) lacks cultural salience in China. Alternate objects for naming tests can be used. Pilot testing of instruments in study samples spanning the full range of diversity in one’s source population can help identify these issues prior to fieldwork. The same considerations would be present clinically, and may be even magnified among those with low levels of education. Based on pilot testing, study investigators may adapt or drop cognitive test items with high rates of missingness, refusal, and/or don’t know (e.g., cactus naming), or that rely on sensory or educational abilities that participants are not guaranteed to have (e.g., vision, hearing, reading, writing, counting).

Exploring cultural considerations and cross-cultural competency is crucial in the assessment and clinical interventions of older individuals with language, fluency and sensory limitations. Healthcare workers, policymakers, and stakeholders should consider the potential clinical implications of differential missingness in cognitive testing due to these and other characteristics. Awareness of cultural considerations and cross-cultural competency is crucial in the assessment and clinical interventions of older individuals with language, fluency, and sensory limitations. Clinicians assessing cognitive decline should be aware that standard cognitive tests may disproportionately exclude individuals with these characteristics, potentially leading to misdiagnoses or underestimation of cognitive impairment. Policymakers should prioritize the development of more inclusive cognitive assessments that accommodate linguistic and sensory diversity, ensuring equitable healthcare access for older adults. Additionally, stakeholders in public health and aging research should invest in strategies to mitigate bias in cognitive testing, such as incorporating multilingual assessments or sensory accommodations. These efforts can improve the accuracy of cognitive evaluations and support informed decision-making in dementia care and policy development.

During data collection in any study, some missing data are inevitable. Missing data, in both research studies and clinical practice, can be avoided through careful piloting of a test battery to identify cross-culturally sensitive measures or measures not otherwise appropriate to a population under study [37]. Approaches to reduce missingness include careful training and booster training for interviewers, and continuous monitoring of data for quality and feedback to field staff. It is also advisable to implement a standard set of missingness categories for every variable that is internally consistent within participants. When missingness is unavoidable, recording such reasons for missingness can help inform missing data mechanisms, which can subsequently aid in deployment of successful imputation strategies. Examples of standard reasons for missing data include don’t know, refusal, skipped or not assessed, computer or interviewer error, cannot read and write, cannot count, physical limitations, language barrier, and uncooperative/poor comprehension. In upcoming waves of the CHARLS-HCAP, investigators are already planning to add additional categories to better capture mechanisms of missingness.

Particularly for large, population-based studies for which data will be used for a multitude of purposes, it can be worthwhile to objectively assess language/dialect of test administration, literacy, and sensory function [38]. For example, subjective, or if possible, objective, measures of vision and hearing can help contextualize non-responses to test items, as indicated by our results. Moreover, these characteristics are also of key scientific interest in gerontological research [39, 40]. Such information can help to contextualize findings and guide scientific inquiries. For example, uncorrected impairment can make visual stimuli used in certain cognitive tests more difficult to see, and auditory stimuli more difficult to hear, which may either impair the respondent’s ability to complete the test or increase the cognitive load required to complete a test [17, 41, 42].

Our study has limitations. Reliable data on primary languages spoken by participants were not collected in CHARLS, and data on language or dialect of interview are not publicly available. Language was imputed by making assumptions based on geographic areas where participants were interviewed; future waves of CHARLS will collect data on language for participants in a more standardized fashion. Relatedly, literacy is self-reported and not objectively tested in the CHARLS study. A limitation regarding DIF detection is that inherent in DIF detection algorithms is an assumption that a relatively small number of tests in a set is presumed to be influenced by an extraneous feature (e.g., language, literacy, sensory impairment). If all or most items show DIF in a constant direction, then no empirical approach can reliably distinguish DIF from real differences in cognitive function. For this reason, the use of anchor items is crucial for DIF analysis. Anchor items are those items that are assumed to have no DIF. In our study, we used a robust sequential-free baseline approach to empirically select anchor items. A final limitation is that coupling the survey data with qualitative interviews or interviewer impressions could have provided insights into the missing data during cognitive testing, which could in turn inform strategies for improving test administration and accessibility. While such qualitative data was not available in the present study, we recommend it for pilot data collection [43].

In sum, CHARLS-HCAP is a large observational cohort study whose cognitive data will be used in many ways, from looking at risk factors for cognitive performance, to imputing data, to operationalizing dementia algorithms. Researchers studying various types of research questions may want to consider different kinds of missing data in different ways. This study has lessons for the adaptation of cognitive interviews such as the HCAP in other countries, training of interviewers, data management, and coding procedures for other population-based studies. There are successful adaptations of common cognitive tests for multilingual populations as well as those with sensory impairment, including those with dual sensory impairment [44–47]. Population-based studies of aging that measure cognition should translate and adapt cognitive test items based on one’s target population for linguistic, cultural, literacy, and sensory impairment needs; reduce missing data during data collection and carefully document reasons for missing data; and assess language or dialect of test administration, literacy, and sensory function of study participants.

## Supplementary Information

Below is the link to the electronic supplementary material.

Supplementary file1 (DOCX 44 kb)

## Data Availability

The data underlying this article are available for download in The China Health and Retirement Longitudinal Study (CHARLS) website, at https://charls.pku.edu.cn/en/. Software code underlying the paper are available from the first author upon request.
