# Supplementary material for: Language, literacy, and sensory impairments and missing cognitive test scores in the Harmonized Cognitive Assessment Protocol of the China Health and Retirement Longitudinal Study
Source: Aging Clin Exp Res. 2025 May 12;37(1):146. doi: 10.1007/s40520-025-03039-y (PMC12069510; doi:10.1007/s40520-025-03039-y)
Supplement: Supplementary file 1 — Supplementary file1 (DOCX 44 kb) [file 40520_2025_3039_MOESM1_ESM.docx]

Supplemental Table 1. DIF by language, literacy, and sensory impairment: Results from CHARLS-HCAP (N=9755)

Legend. Odds Ratios (OR) illustrate the difference (on an odds scale) in cognitive test performance between each grouping feature, adjusting for the latent cognitive ability. An OR>1.0 suggests the item is more likely to be correct in the focal group compared to the reference group, controlling for underlying cognition.

Supplemental Table 2. Number of participants with cognitive factor scores with salient DIF according to language, literacy, and sensory impairment: Results from CHARLS-HCAP (N=9755)

| Feature | N (%) |
| --- | --- |
| Likely Mandarin (reference: Confirmed Mandarin) | 665 (6.8%) |
| Likely Non-Mandarin (reference: Confirmed Mandarin) | 0 (0%) |
| Illiterate (reference: literate) | 10 (0.1%) |
| Hearing impairment (reference: Hearing unimpaired) | 0 (0%) |
| Vision impairment (reference: Vision unimpaired) | 0 (0%) |

Legend. Salient DIF was calculated as the difference between DIF-adjusted and non-DIF-adjusted factor scores, for each grouping variable. The number of participants whose DIF-adjusted scores differed by more than 0.3 standard deviation units from non-DIF-adjusted scores are shown.
